# Prevalence of elevated serum anti-N-methyl-D-aspartate receptor antibody titers in patients presenting exclusively with psychiatric symptoms: a comparative follow-up study

**DOI:** 10.1186/s12888-016-0948-9

**Published:** 2016-07-08

**Authors:** Yoshihito Ando, Haruo Shimazaki, Katsutoshi Shiota, Syuichi Tetsuka, Koichi Nakao, Tatsuhiro Shimada, Kazumi Kurata, Jinichi Kuroda, Akihiro Yamashita, Hayato Sato, Mamoru Sato, Shinkichi Eto, Yasunori Onishi, Keiko Tanaka, Satoshi Kato

**Affiliations:** Department of Neurology, Haga Red Cross Hospital, 2461 Daimachi, Moka, Tochigi 321-4306 Japan; Division of Neurology, Department of Internal Medicine, Jichi Medical university, Shimotsuke, Tochigi Japan; Department of Psychiatry, Jichi Medical University, Shimotsuke, Tochigi Japan; Department of Neurology, International University of Health and Welfare Hospital, Nasushiobara, Tochigi Japan; Department of Neurology, Junwakai Memorial Hospital, Miyazaki, Japan; Department of Psychiatry, Tochigi Prefectural Okamotodai Hospital, Utsunomiya, Tochigi Japan; Department of Psychiatry, Sato Hospital, Yaita, Tochigi Japan; Department of Psychiatry, Kamitsuga General Hospital, Kanuma, Tochigi Japan; Department of Psychiatry, Oyama Fujimidai Hospital, Shimotsuke, Tochigi Japan; Department of Life Science, Medical Research Institute and Department of Neurology, Kanazawa Medical University, Uchinada, Ishikawa Japan

**Keywords:** Psychiatric symptom, Schizophrenia, Anti-N-methyl-D-aspartate receptor antibody, Human embryonic kidney 293 cell, Prospective study

## Abstract

**Background:**

Increasing numbers of patients with elevated anti-N-methyl-D-aspartate (NMDA) receptor antibody titers presenting exclusively with psychiatric symptoms have been reported. The aim of the present study was to clarify the prevalence of elevated serum anti-NMDA receptor antibody titers in patients with new-onset or acute exacerbations of psychiatric symptoms. In addition, the present study aimed to investigate the association between elevated anti-NMDA receptor titers and psychiatric symptoms.

**Methods:**

The present collaborative study included 59 inpatients (23 male, 36 female) presenting with new-onset or exacerbations of schizophrenia-like symptoms at involved institutions from June 2012 to March 2014. Patient information was collected using questionnaires. Anti-NMDA receptor antibody titers were measured using NMDAR NR1 and NR2B co-transfected human embryonic kidney (HEK) 293 cells as an antigen (cell-based assay). Statistical analyses were performed for each questionnaire item.

**Results:**

The mean age of participants was 42.0 ± 13.7 years. Six cases had elevated serum anti-NMDA antibody titers (10.2 %), four cases were first onset, and two cases with disease duration >10 years presented with third and fifth recurrences. No statistically significant difference in vital signs or major symptoms was observed between antibody-positive and antibody-negative groups. However, a trend toward an increased frequency of schizophrenia-like symptoms was observed in the antibody-positive group.

**Conclusion:**

Serum anti-NMDA receptor antibody titers may be associated with psychiatric conditions. However, an association with specific psychiatric symptoms was not observed in the present study. Further studies are required to validate the utility of serum anti-NMDA receptor antibody titer measurements at the time of symptom onset.

## Background

Anti-N-methyl-D-aspartate (NMDA) receptor encephalitis with a characteristic clinical course was proposed in 2007 by Dalmau et al. [1]. The disease classically progresses from flu-like to schizophrenia-like symptoms, leading to intractable convulsions, involuntary movements, and ultimately, an unresponsive state requiring artificial respiration management for central hypoventilation. However, many severe cases will make a full recovery [1]. Approximately half of affected patients are found to have an ovarian teratoma with early tumoral excision reported to be associated with improved recovery phase. Administration of immunomodulating drugs, including adrenal corticosteroids, large doses of intra-venous immunoglobulins, and plasma exchange, represents the first-line treatment for anti-NMDA receptor encephalitis. Immunosuppressive drugs, including cyclophosphamide and rituximab, are recommended as second-line treatments [2].

However, there has been a recent increase in reports of anti-NMDA receptor encephalitis in patients without ovarian tumors, including male and pediatric cases.

We previously reported the case of an 18-year-old man with anti-NMDA receptor antibodies who developed psychiatric symptoms following type B influenza virus infection. The patient did not develop severe symptoms such as convulsions or central hypoventilation and experienced a full recovery following modified electroconvulsive therapy for psychiatric symptoms.

Anti-NMDA receptor encephalitis is often managed by psychiatry departments as patients typically present exclusively with psychiatric symptoms, as in the present case. The prevalence of anti-NMDA receptor antibodies has previously been reported; however, this was a retrospective study that predominantly used preserved specimens.

We prospectively examined cases with the development of new-onset or exacerbations of psychiatric symptoms to assess the prevalence of anti-NMDA receptor antibody positivity and assess its association with psychiatric symptoms in a multi-center collaborative study.

## Methods

### Study setting and subjects

The present study included inpatients that developed new-onset or exacerbations of schizophrenia-like symptoms and were from neurology and psychiatry departments at institutions from June 2012 to March 2014. Target cases were chosen by the study partaker of each institution. When agreement was not provided by the patients themselves, we explained the purpose of the present study to a family member and obtained their consent. We obtained written consent from patients and/or their families (caregivers) when patients were unable to comprehend because of psychiatric symptoms. Furthermore, written consent to publication of more detailed clinical information such as age, sex, and symptoms was included.

The present study was conducted with the approval of the Bioethics Committee for Clinical Research, Jichi Medical University Hospital. Any collaborating institution without its own ethical review board was also represented by the Bioethics Committee for Clinical Research, as requested in writing.

A total of 59 cases (23 male, 36 female) were enrolled. The mean age was 42.0 ± 13.7 (SD) years. Twenty-one patients had a first episode of symptoms, 37 had recurrent episodes (second, third, fourth, and fifth recurrences in 19, five, three, and ten patients, respectively), and one case was unclear.

Patient information was collected using a questionnaire completed by participants. The initial questionnaire at the time of the first examination included questions regarding age, sex, blood pressure, pulse rate, body temperature, and Japan coma scale (JCS). Further sections were as follows: clinical diagnosis according to Diagnostic and Statistical Manual of Mental Disorders IV text revision (DSM-IV TR); history of preceding infection; characteristics of onset or recurrence; number of recurrences and disease duration; psychiatric symptoms (anxiety/depression, mania, or schizophrenia-like symptoms); and neurological symptoms. Furthermore, we included items related to psychiatric symptoms and neurological symptoms (Table 1). The JCS is the original Japanese consciousness scale and is more widely used than the Glasgow coma scale (GCS) in Japan. However, the accurate comparison of scores between the GCS and JCS is challenging as there is no item corresponding to the “Verbal” score in the JCS. A consciousness score of 0 indicates the patient is lucid, and scores of 1–3 are equivalent to a GCS of E4, indicating spontaneous eye-opening. Scores of 10–30 indicate eye-opening in response to stimulation, with a score of 10 being equivalent to a GCS score of E3 and scores of 20–30 being equivalent to E2. Scores >100 are equivalent to E1, and a score of 300 is equivalent to a score of M1 on the GCS.

**Table 1:** Study questionnaire

ID: Initial: Sex: Age:
Blood pressure, Pulse rate, Body temperature
Japan coma scale
(1) DSM-IV diagnosis:
(2) Precedent infectious symptoms (fever/headache/throat pain/diarrhea)
(3) The first episode or the exacerbation
3–1) How much time had passed from the onset of symptoms until receiving a medical examination?
3–2) How long have you been under psychiatric care in the hospital?
less than 1 month/less than 3 months/less than 1 year
less than 5 years/less than 10 years/more than 10 years
3–3) How many times did your symptoms exacerbate?
two/three/four/five/more than five
(4) Psychiatric symptom(s) (possible plural answers)
a) anxiety/depression symptom
irritation/anxiety/depressive mood/depersonalization
sleep disorder/will drop/concentration drop/suicidal feeling
b) mania symptom
hyperthymia/hyperlogia/hyperactivity/flight of idea
c) schizophrenia-like symptom
auditory hallucination/visual hallucination/delusion/obfuscation
excitement/echolalia/echopraxia/stereotypic language
stereotypic movement/catalepsy/idle–autosynnoia/flattened feeling
(5) Neurological symptom(s)
convulsions/orolingual dyskinesia/systemic dyskinesia/eyeball dyskinesia
instability of blood pressure and pulse/sweat and drooling abnormality
respiratory depression/disorientation/amnesia

### Statistical analyses

Patients were divided into two groups on the basis of the presence or absence of anti-NMDA receptor antibodies, and answers to individual questionnaire items were compared. Welch’s *t*-test was performed for comparison of continuous variables, including age, blood pressure, pulse rate, and body temperature. For categorical data such as sex and items assessing the presence or absence of symptoms, Fisher’s direct exact test was used because of the small number of antibody-positive patients. JCS at the time of the hospitalization was divided into seven classes: 0, 1, 5, 3, 10, 30, and 100. The number of recurrences was divided into five classes: 1, 2, 3, 4, and >5. Disease duration was divided into seven classes: first, 1 month, 3 month, 1 year, 5 years, 10 years, and >10 years, which were assessed using the Mann–Whitney *U* test. All statistics were performed using the software “Stat Flex ver.6”(Artech, Osaka, Japan). All tests were two-tailed, and significance was set at *P* < 0.05.

### NMDA receptor antibody measurements

Blood and/or cerebrospinal fluid was collected at the time of hospitalization at each institution. Serum was extracted, frozen, and stored until further use. All samples were anonymized and sent to the Department of Neurology, Jichi Medical University. A frozen serum specimen for each patient was subsequently sent to the Department of Neurology, Kanazawa Medical University.

All serum obtained at the first study visit was measured using the following methods.

**Fig. 1:**
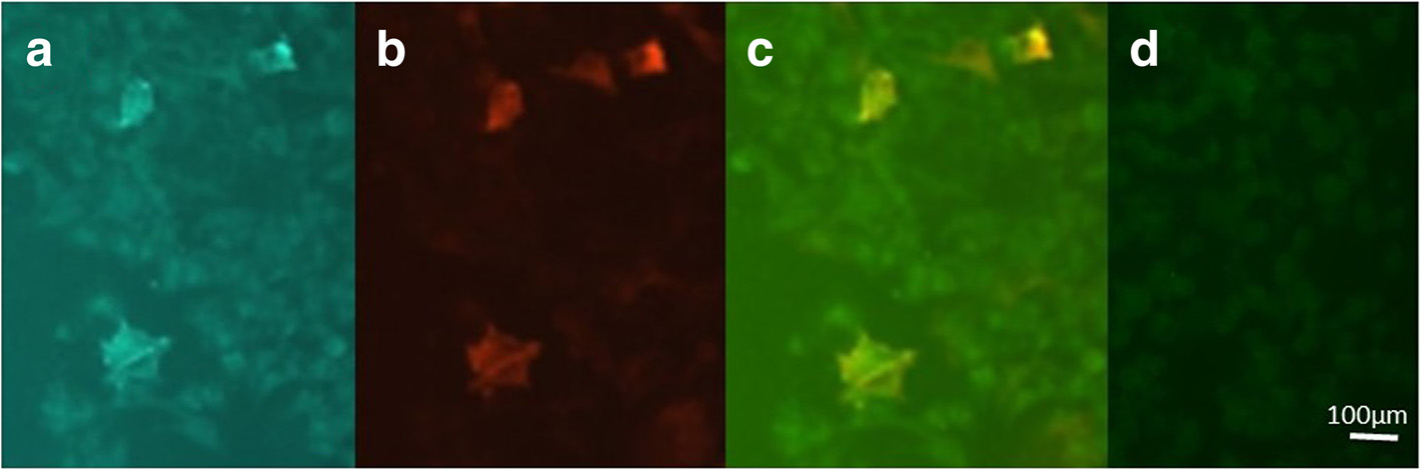
Immunohistochemical analyis of HEK cells co-expressing NR1 and NR2B subunits. HEK cells were incubated in patient serum (1:20 dilution) and rabbit anti-NR1 antibody (1:100 dilution). **a** FITC-conjugated anti-human IgG visualized as blue fluorescence. **b** PE-conjugated anti-rabbit IgG visualized as red fluorescence. **c** Co-localization of blue and red fluorescence indicating reaction to the same epitope (NR1 subunit). **d** Negative control (HEK cells transfected with empty vector)

Human embryonic kidney (HEK) 293 cells transfected with plasmid DNAs encoding the NMDA receptor subunits NR1 and NR2B with cell membrane co-expression were used [3]. Cell culture was maintained in Dulbecco’s modified Eagle's medium with 10 % fetal calf serum supplemented with 10 μM MK-801 (Wako, Tokyo, Japan) for neuroprotection. At 12 h after transfection, HEK cells were fixed in 4 % paraformaldehyde in 0.1 M phosphate-buffered saline (PBS, pH 7.4) for 20 min. Non-specific binding was blocked with 10 % goat serum/PBS, and cells were incubated with patient sera (1:10–400) in 0.02 % Triton X-100 and 10 % goat serum in PBS overnight at 4 °C and then with fluorescein isothiaocyanate (FITC)-conjugated anti-human IgG (DAKO, Glostrup, Denmark; 1:50) and phycoerythrin (PE)-conjugated anti-rabbit IgG secondary antibodies (DAKO, Glostrup, Denmark; 1:50) for 1 h. SlowFade Gold anti-fade reagent (Invitrogen Japan, Tokyo) was then applied to the slides, and staining was observed under a fluorescence microscope (Axiovision, Zeiss). To confirm the localization of NMDAR antibody-binding sites, double staining was performed with both patient samples and a mixture of rabbit anti-NR1 (PhosphoSolutions, CO, USA; 1:10) and anti-NR2B (Frontier Science, Sapporo, Japan; 1:10) antibodies. Then, primary antibodies were visualized by FITC-anti-human IgG and R-PE-anti-rabbit IgG and observed under a laser scanning microscope (Zeiss LSM 710). IgG binding was confirmed as IgG1. No staining was observed in HEK cells expressing homomeric NR2B subunits or transfected with an empty vector. Serial dilutions were used, and where co-localization was observed, antibody titers were measured (Fig. 1). Serum from subsequent follow-up visits was analyzed using the same methodology.

### Immunohistochemistry

Paraffin-embedded sections of mouse brain (Genostaff Co. Ltd. Tokyo, Japan) were stained with rabbit anti-GluN1 antibody and anti-GluN2B antibody (Frontier Institute Co. Ltd. Hokkaido, Japan) at a 1:200 dilution or human serum at a 1:1000 dilution that included the serum of an antibody-positive 59-year-old male patient. Staining was detected using biotinylated anti-rabbit or anti-human Ig secondary antibodies (Vector Laboratories, Inc., Burlingame, CA, USA). For histochemical evaluation, hematoxylin staining was used to visualize the overall structure of each section.

### Animal experiments

Animal experiments were conducted in a humane manner after receiving approval from the Institutional Animal Experiment Committee of the GENOSTAFF CO.LTD. and in accordance with the Institutional Regulation for Animal Experiments and Fundamental Guideline for Proper Conduct of Animal Experiment and Related Activities in Academic Research Institutions under the justification of the Ministry of Education, Culture, Sports, Science and Technology.

## Results

Six out of 59 cases had detectable serum anti-NMDA antibody titers (10.2 %). The clinical diagnosis at the time of initial examination in anti-NMDA receptor antibody patients was schizophrenia in four cases, major depression in one case, and undetermined in one case. Four cases were new onset, and the other two cases had a disease duration of greater than 10 years, with third and fifth recurrences.

Clinical features of NMDA antibody-positive and antibody-negative patients are shown in Table 2. With regard to episode recurrence, initial and recurrent episodes were observed in 53 antibody-negative patients, and no difference in the proportion of recurrent episodes was observed between the antibody-negative and antibody-positive groups by the Mann–Whitney test (*P* = 0.329). In addition, no significant difference in disease duration was observed between groups (*P* = 0.499).

**Table 2:** Clinical history of NMDA antibody-positive patients

Age/Sex	Diagnosis	Disease duration	Exacerbation number of times	First examination		Second examination				Third examination
				Symptom	Dilution titer of antibody	Symptom	Duration from onset	Dilution titer of antibody	Result of CT scan	
59/M	Major depression	>10 years	3	Delusion, Anxiety, Insomnia, Irritation, Suicidal feeling, Excitement, Obfuscation	×1000	None	7 months	<×2 (Negative)	Bilateral renal cysts	Not examined
22/F	Schizophrenia	0	1	Auditory hallucination, Oral dyskinesia	×10	Amelioration (auditory hallucination, delusion)	3 months	<×2 (Negative)	No tumor	Not examined
56/F	Schizophrenia	0	1	Catalepsy, Irritation, Insomnia, Polyphrasia	×10	None	5 months	<×2 (Negative)	No tumor	Not examined
44/M	Schizophrenia	0	1	Auditory hallucination, Delusion, Anxiety, Flattened feeling	×20	Amelioration (Tonic state/development disorder)	5 months	×5 (Positive)	No tumor	On 11 months from onset, tonic symptom remains, antibody dilution titer < ×2
22/F	Schizophrenia	>10 years	5	Auditory hallucination, Delusion, Excitement	×20	Amelioration (delusion of persecution)	7 months	<×2 (Negative)	No tumor	Not examined
47/F	Undetermined → Schizophrenia	0	1	Excitement, Catatonia, Obfuscation	×20	None	6 months	×10 (Positive)	Myoma of uterus	Not examined

The mean age of patients in the antibody-positive and antibody-negative groups was 41.7 ± 16.2 and 41.9 ± 13.5 years, respectively, with no significant difference being observed between groups (*P* = 0.977). There was a higher proportion of females in the study overall, with ratios of 60.4 and 66.7 % in antibody-negative and antibody-positive groups, respectively. No significant difference in gender was observed between groups (*P* = 1.000).

No significant differences in systolic blood pressure (*P* = 0.648), diastolic blood pressure (*P* = 0.578), or body temperature (*P* = 0.547) were observed between groups at the time of the first examination; however, there was a trend toward a higher pulse rate in the antibody-negative group than in the antibody-positive group (96.7/min vs. 80.3/min, *P* = 0.0971).

With regard to the consciousness level at the time of the first examination, the majority of patients were either lucid or slightly confused; however, a number of patients in the antibody-negative group presented with somnolence or semi-coma. No significant difference in the numerical distribution of JCS scores was observed between groups (*P* = 0.424).

No differences in the frequency of preceding infections (*P* = 0.581) or the proportion of patients reporting depression/anxiety symptoms (*P* = 0.382), mania symptoms (*P* = 0.660), convulsions (*P* = 1.000), involuntary movements (*P* = 1.000), blood pressure and pulse rate fluctuations (*P* = 1.000), dyshydrosis (*P* = 1.000), or amnesia (*P* = 0.594) were observed between groups.

However, there was a trend toward a greater proportion of antibody-positive patients reporting schizophrenia-like symptoms (*P* = 0.198). Furthermore, there was a slight trend toward reporting of blunting of feelings (*P* = 0.362), delusion (*P* = 0.323), and obfuscation (*P* = 0.577) in antibody-positive patients. No significant differences in any other lower-rank items were observed between groups.

Neurological symptoms such as confusion, dyskinesia, sweating, and amnesia have been reported by psychiatrists (not neurologists) in seronegative patients. Accordingly, the term “psychiatric symptom cases” may not be entirely appropriate as symptoms were very mild compared with those of NMDA encephalitis, and neurological symptoms are rarely reported in antibody-positive cases.

The six antibody-positive patients were followed up for a mean duration of 5.5 ± 1.4 months. Psychiatric symptoms resolved in three cases, and the other three cases entered a state of amelioration. Oral medication was maintained in all cases (Table 3). Thoracoabdominal-enhanced computed tomographic imaging was performed in all cases for tumor surveillance. One case of uterine myoma was identified in a female patient. No ovarian teratomas, seminomas, or germinomas were detected in any cases. Furthermore, we measured serum tumor marker levels, which were all within the normal range in all cases, expect one case with a slightly elevated neuron-specific enolase (NSE) level.

**Table 3:** Result of statistical analysis

	Positive patients (*n* = 6)	Negative patients (*n* = 53)	*P*-value	Statistical method
Age	41.67 ± 16.21	41.89 ± 13.54	0.9775	Welch’s *t* test
Sex (M/F)	2/4	21/32	1.0000	Fisher’s direct exact test
Pulse rate (/min)	80.33 ± 17.42	96.66 ± 21.35	0.0971	Welch’s *t* test
Systolic blood pressure (mmHg)	122.17 ± 19.71	126.42 ± 20.83	0.6483	Welch’s *t* test
Diastolic blood pressure (mmHg)	78.17 ± 13.53	81.76 ± 14.51	0.5782	Welch’s *t* test
Body temperature (°C)	36.55 ± 0.65	36.74 ± 0.46	0.5471	Welch’s *t* test
Japan coma scale (0/1/2/3/10/30/100)	4/2/0/0/0/0/0	19/12/5/3/1/1/1	0.4245	Mann–Whitney *U* test
Precedent infection	0	8	0.5812	Fisher’s direct exact test
Episode times (1/2/3/4/>5)	4/0/1/0/1	17/19/4/3/9	0.3291	Mann–Whitney *U* test
Treatment period (0/3 months/1 years/5 years/10 years/>10 years)	4/0/0/0/0/0/2	18/1/1/2/11/8/10	0.4987	Mann–Whitney *U* test
Anxiety/Depression symptoms	3	36	0.3822	Fisher’s direct exact test
Irritation	3	23	1.0000	
Anxiety	2	24	0.6814	
Depressive mood	1	6	1.0000	
Depersonalization	0	0	*	
Sleep disorder	2	25	0.6752	
Will drop	1	5	1.0000	
Concentration drop	1	8	1.0000	
Suicidal feeling	1	8	1.0000	
Mania symptoms	1	16	0.6597	
Hyperthymia	1	10	1.0000	
Hyperlogia	1	11	1.0000	
Hyperactivity	0	4	1.0000	
Flight of idea	0	2	1.0000	
Schizophrenia-like symptoms	5	51	0.1978	
Auditory hallucination	3	31	0.6838	
Visual hallucination	0	3	1.0000	
Delusion	3	40	0.3231	
Obfuscation	2	8	0.5770	
Excitement	3	24	1.0000	
Echolalia	0	0	*	
Echopraxia	0	3	1.0000	
Stereotypic language	0	0	*	
Stereotypic movement	0	0	*	
Catalepsy	0	4	1.0000	
Idle–Autosynnoia	0	4	1.0000	
Flattened feeling	1	3	0.3619	
Neurological symptoms	0	1	1.0000	
Convulsion (Complex partial seizure)	0	1	1.0000	
Orolingual dyskinesia	1	2	0.2838	
Systemic dyskinesia	0	1	1.0000	
Eye ball dyskinesia	0	2	1.0000	
Instability of blood pressure	0	1	1.0000	
Sweating and drooling abnormality	0	3	1.0000	
Disorientation	0	0	*	
Amnesia	0	7	0.5739	

* statistical examination was not performed

We measured serum titers of anti-NMDA antibodies in the six antibody-positive patients at the second visit. In four of the six cases, antibody levels became undetectable at a twofold dilution. The other two cases remained positive at the second visit; however, titers were reduced from the first visit in both patients. One case was a 44-year-old man who presented with onset of delusion, auditory hallucination, and anxiety. He was diagnosed with developmental disability in a hypertonic state at the time of the follow-up survey 5 months later. The serum antibody titer was detectable at a 20-fold dilution at the first visit and remained detectable at a fivefold dilution at the second visit. Serum titers became negative another 11 months later; however, he remained symptomatic.

The other case was a 47-year-old woman who was initially undiagnosed before receiving a diagnosis of schizophrenia according to the DSM-IV TR. Her psychiatric symptoms began with the onset of excitement and catatonic substupor, with initial serum antibody titers being detectable at a 20-fold dilution. The patient demonstrated no apparent symptoms at the follow-up survey 6 months later; however, serum antibody titers remained detectable at a 10-fold dilution. Following this visit, the patient discontinued her medication, leading to recurrence of obfuscation and auditory hallucination symptoms 8 months following the first episode. She was hospitalized in a mental hospital for 2 months and received medical therapy thereafter. No symptoms were apparent 16 months after the first episode. However, we were unable to obtain her final serum antibody titer.

**Fig. 2:**
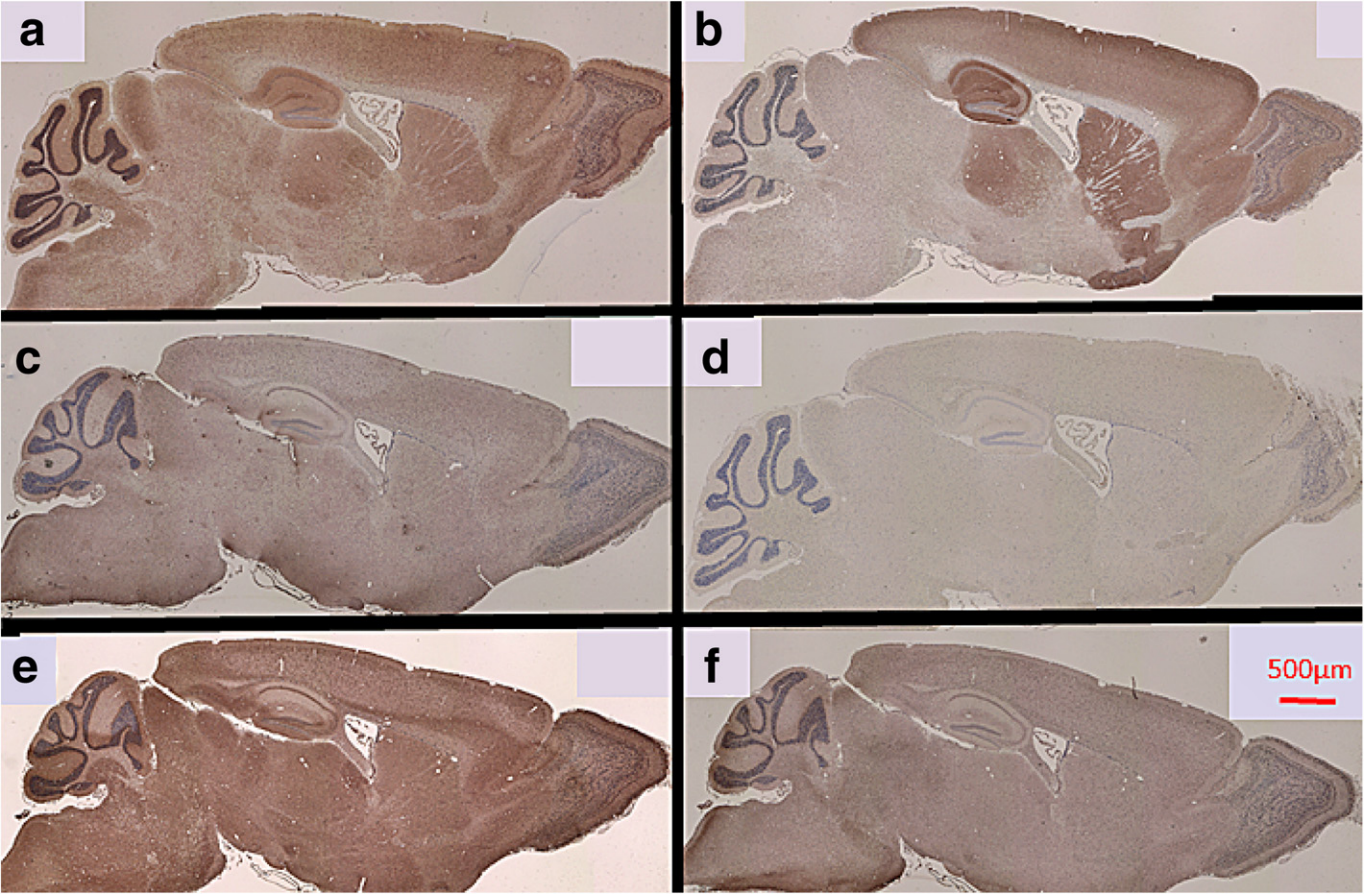
Immunohistochemical analysis of paraffin-embedded mouse brain specimens. **a** The cerebral cortex, basal nuclei, and cerebellum were widely stained by anti-GluN1 (NR1) antibody at a 200-fold dilution. **b** Particularly strong staining of the hippocampus and basal nuclei was observed with the anti-GluN2B (NR2B) antibody at a 200-fold dilution. The characteristic staining pattern was not observed with healthy control serum at a 1000-fold dilution (**c**) and with nonspecific IgG at a 200-fold dilution (**d**). **e** Representative images of extensive staining with anti-GluN1 from the serum of a 59-year-old male patient at onset at a 1000-fold dilution. **f** The characteristic stain was not observed with the use of serum from a healthy control patient whose symptoms improved at a 1000-fold dilution

For comparison, we performed immunohistochemical analysis of paraffin-embedded mouse brain specimens. Antibodies against the NR1 and NR2B subunits of the NMDA receptor were used as positive controls. The cerebral cortex, cerebellum, hippocampus, and basal nucleus were stained by the NR1 antibody, and the hippocampus and basal nucleus were stained particularly strongly by the NR2B antibody. No staining was observed using the serum of healthy controls or non-specific IgG serum as a negative control. The cerebral cortex, cerebellum, hippocampus, and basal nucleus were stained by the serum of a 59-year-old man who was anti-NMDA receptor antibody-positive at the time of the initial examination. The staining pattern was similar to that observed with the NR1 antibody. Serum obtained when the patient had become antibody-negative 7 months after the first episode demonstrated no staining, with comparable findings to those observed with healthy control serum (Fig. 2).

## Discussion

The NMDA receptor antibody was first reported by Dalmau et al. as the antibody responsible for autoimmune encephalitis in young women with ovarian tumors, which follows a characteristic clinical course. It has subsequently become clear that elevation of NMDA receptor serum titers is associated with variable clinical courses [1, 2].

The use of anti-NMDA receptor antibody measurement has recently attracted great attention in the field of psychiatry. In addition, there are many reports of the association between elevations of serum NMDA receptor antibody levels and various diseases presenting with psychiatric symptoms.

Rhoads et al. [4], Haussleiter et al. [5], and Masdeu et al. [6] reported no cases of elevated serum anti-NDMA receptor antibody titers in patients with schizophrenia; however, Zandi et al. reported elevated levels of 6.5 % in patients with early-phase psychiatric disorder [7], and Tsutsui et al. reported a prevalence of 7.8 % in patients with schizophrenia. Furthermore, a high prevalence of elevated titers of 60 % has been reported in narcoleptic patients with psychiatric symptoms [8]. Steiner et al. reported elevated titers in 9.9 % of patients with schizophrenia and 2.8 % of patients with major depression in 2013 [9]. Hammer et al. evaluated the preserved serum of more than 1000 patients with schizophrenia and reported a prevalence of 8.6 % [10]. In the present study, the prevalence of elevated anti-NMDA receptor antibody titers was 10.2 %, and it was found to be 9.1 % in patients initially diagnosed with schizophrenia. This prevalence is in accordance with previous reports (Table 4).

**Table 4:** Comparison of previous reports

	n	M/F	Diagnosis	Positive	Prevalence	Antibody measure method
Rhoads et al. 2011 [4]	7	3/4	Schizophrenia	0	0.0 %	Cell-based assay NR1
Zandi et al. 2011 [7]	46	ns	Early-stage psychiatric disorder	4	6.5 %	Cell-based assay NR1/NR2B
			Schizophrenia 63 %			
			Atypical psychosis 15 %			
			Mood disorder 4 %			
			Bipolar disorder 4 %			
			Major depression 2 %			
			Paranoia 2 %			
Haussleiter et al. 2012 [5]	50	17/33	Psychiatric disorder	0	0.0 %	Cell-based assay NR1/NR2B, NR1/NR1
Masdeu et al. 2012 [6]	80		Schizophrenia	0	0.0 %	Cell-based assay NR1
Tsutsui et al. 2012 [8]	51	ns	Schizophrenia	4	7.8 %	Cell-based assay NR1/NR2B
	5	ns	Narcolepsy with psychiatric symptom	3	60.0 %	
Steiner et al. 2013 [9]	121	77/44	Schizophrenia	12	9.9 %	Cell-based assay NR1/NR2B
	70	26/44	Major depression	2	2.8 %	
	38	14/24	Borderline personality disorder	0	0.0 %	
Hammer et al. 2014 [10]	1272	780/492	Healthy control	137	10.8 %	Cell-based assay NR1/NR2B
	1081	723/358	Schizophrenia	93	8.6 %	
	148	70/78	Affective disorder	24	16.2 %	
Steiner et al. 2014 [11]	357	ns	Healthy control	25	7.0 %	Indirect immunofluorescence BIOCHIP assay (commercial)
	184	ns	Schizophrenia	18	9.8 %	
	99	ns	Major depression	5	5.1 %	
	42	ns	Borderline personality disorder	1	2.4 %	
Our report 2015	59	23/36	Total	6	10.2 %	Cell-based assay NR1/NR2B
	44	15/29	Schizophrenia	4	9.1 %	
	1	1/0	Major depression	1	100.0 %	
	4	1/3	Undetermined	1	25.0 %	
	1	1/0	Nonherpetic limbic encephalitis	0	0.0 %	
	1	0/1	Atypical psychosis	0	0.0 %	
	4	1/3	Acute transient psychosis	0	0.0 %	
	1	1/0	Bipolar disorder	0	0.0 %	
	2	1/1	Organic psychosis	0	0.0 %	
	1	1/0	Mental retardation	0	0.0 %	

Elevated titers were observed in cases with third and fifth recurrences greater than 10 years after the initial diagnosis and in new-onset cases. Previous studies have retrospectively measured antibody titers using preserved serum and performed clinical examination of seropositive patients to assess symptoms; however, these studies were unable to follow-up with patients and assess later changes in antibody titers and symptoms.

We prospectively examined new inpatients, in contrast to previous studies, and followed-up with seropositive patients. Furthermore, we demonstrated the normalization of elevated serum anti-NMDA receptor antibody titers with symptom improvement in five of six seropositive cases. This result indicates the anti-NMDA receptor antibody may influence the progression of psychiatric symptoms.

However, our findings raise two important issues regarding this hypothesis. First, antibody titers remained elevated in two cases despite the resolution of psychiatric symptoms. Second, when comparing major symptoms at the time of onset in antibody-positive patients against antibody-negative patients, we observed no statistically significant differences in the frequency of different symptoms between the two groups.

In addition, Hammer et al. reported the prevalence of elevated serum anti-NMDA receptor antibody titers to be 10.8 % in healthy controls, which is higher than the value of 8.6 % observed in preserved specimens from schizophrenia cases [10]. Steiner et al. reported the prevalence of elevated titers to be 7.0 % in healthy control serum by supplementary examination in 2014 [11]. Further, Steiner et al. posited that this increase in the reported prevalence was likely due to differences in measurement method between commercial kits; however, Hammer et al. utilized a HEK cell-based assay, as described previously [10]. Interestingly, recent evidence has indicated a constant prevalence of elevated titers in the healthy general population.

A control group was not included in this study because we initially aimed to compare symptoms of patients between the presence or absence of anti-NMDA antibodies. However, a control group could have been useful for the interpretation of results, using the same method of data comparison between a control group and past records of healthy subjects.

Maneta et al. posited that psychiatric symptoms develop only when environment factors such as increased cytokine levels in association with schizophrenia affect the blood–brain barrier allowing anti-NMDA receptor antibody present in the serum to enter the central nervous system, thereby leading to the development of symptoms [12].

In typical NMDA receptor encephalitis, the mechanism underlying symptom onset is more apparent as the immune system is activated by preceding infection in the presence of autoantibodies against ovarian teratoma. However, increasing numbers of atypical cases without preceding infection or ovarian teratoma, as described in the original typical cases, are being reported. Thus, the determination of causative environmental factors in the present antibody-positive cases that have neither tumors nor preceding infection is particularly challenging.

Maneta et al. described a correlation between anti-NMDA receptor antibody titers and the severity of symptoms [12]. We followed-up symptom severity and antibody titers in antibody-positive patients. Antibody titers decreased in parallel with symptomatic improvements in six antibody-positive patients included in the present study. In the other two cases that remained positive at the second visit, antibody titers decreased from the first visit in both patients. However, antibody titers in the asymptomatic female case were higher than those in the symptomatic male case (10-fold vs. fivefold). This finding indicates that individual changes in antibody titers may reflect a disease condition; however, the measurement of antibody titers has limited utility in disease conditions between multiple patients. Future studies are required to validate the utility of determining the absence of symptom recurrence in confirming the antibody-negative status and clarifying the association between antibody titer levels and symptomatic changes.

Cerebrospinal fluid (CSF) anti-NMDAR antibody titers are thought to provide a more accurate reflection of encephalitis symptoms than serum titers. Furthermore, our results corroborate the findings of a previous study reporting that CSF and serum antibody titers tend to decrease with amelioration of symptoms, even if titer levels do not become entirely negative [13]. In addition, differences in reported symptoms according to antibody subclass have previously been discussed [9, 11].

As the present study was conducted in psychiatric settings, the collection of CSF was technically challenging, and we were able to measure serum IgG antibody levels only. Further, serum and CSF antibody titer levels may not always change in parallel. Therefore, results may differ according to the use of either CSF or serum antibody titer measurements.

Moreover, no patients received immunotherapy as the patients included in the present study were diagnosed with psychiatric disorders such as encephalitis and admitted to a psychiatric hospital. Previous studies have reported the immunosuppressive effect of antipsychotics, particularly dopamine D2 receptor antagonist, in animal experiments [14]. Although detailed information regarding patient prescription contents were unavailable, many patients were treated with antipsychotic drugs, including dopamine D2 receptor antagonists. Accordingly, the immunosuppression effect of such drugs may have influenced the disease condition.

However, in light of recent studies, it is apparent that NMDA receptor antibodies exert an effect on the onset of psychiatric disease. In our study, we measured antibody titer using a HEK cell-based assay designed “in-house”. This method required specialized techniques and specific resources, i.e, our approach may prove to be challenging to repeat elsewhere; thus, it lacks universal reproducibility. Unified methods for the measurement of serum anti-NMDA receptor titers, ideally simple and easy to perform, are required for determining cut-off thresholds and assessing the potential utility of antibody titer measurements in the surveillance of psychiatric illnesses.

## Conclusion

Anti-NMDA receptor serum antibody was detected in 10.2 % of new-onset and recurring cases of acute psychiatric symptoms in the present study. Serum antibody titers may rise during symptom development, not only during the initial onset but also during recurrent episodes. Further, a change in antibody titers may predict the clinical condition in individual patients. However, no statistical differences in clinical symptoms or patient background according to the presence of anti-NMDA antibodies were observed in the present study. Furthermore, the contribution of anti-NMDA encephalitis to psychiatric symptom onset and the pathological significance of elevated antibody titers remain unclear. Further studies and the accumulation of case studies are required to increase understanding of the pathological significance of anti-NMDA receptor antibodies.

## Abbreviations

CSF, cerebrospinal fluid; DSM-IV TR, Diagnostic and Statistical Manual of Mental Disorders IV text revision; FITC, fluorescein isothiaocyanate; GCS, Glasgow Coma Scale; HEK, human embryonic kidney; JCS, Japan coma scale; NMDA, N-methyl-D-aspartate; PBS, phosphate-buffered saline; PE, phycoerythrin.
